# Hyperpigmented papule in patient with breast cancer history

**DOI:** 10.1016/j.jdcr.2024.11.034

**Published:** 2024-12-08

**Authors:** Maya Akbik, Mia S. DeSimone, Vinod E. Nambudiri

**Affiliations:** a Medical College of Georgia, AU/UGA Medical Partnership, Athens, Georgia; b Department of Dermatology, Brigham and Women's Hospital, Boston, Massachusetts; c Department of Pathology, Brigham and Women’s Hospital, Harvard Medical School, Boston, Massachusetts

**Keywords:** biopsy, hyperpigmented papule, metastatic breast cancer, vascular lesion

## History

**Fig 1:**
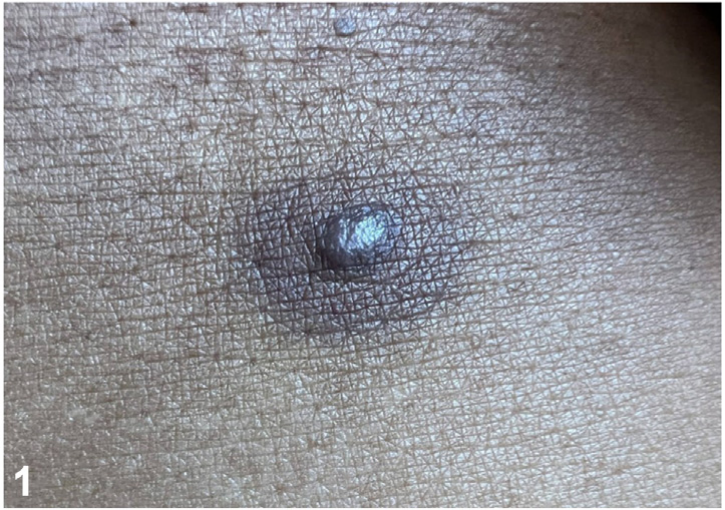

**Fig 2:**
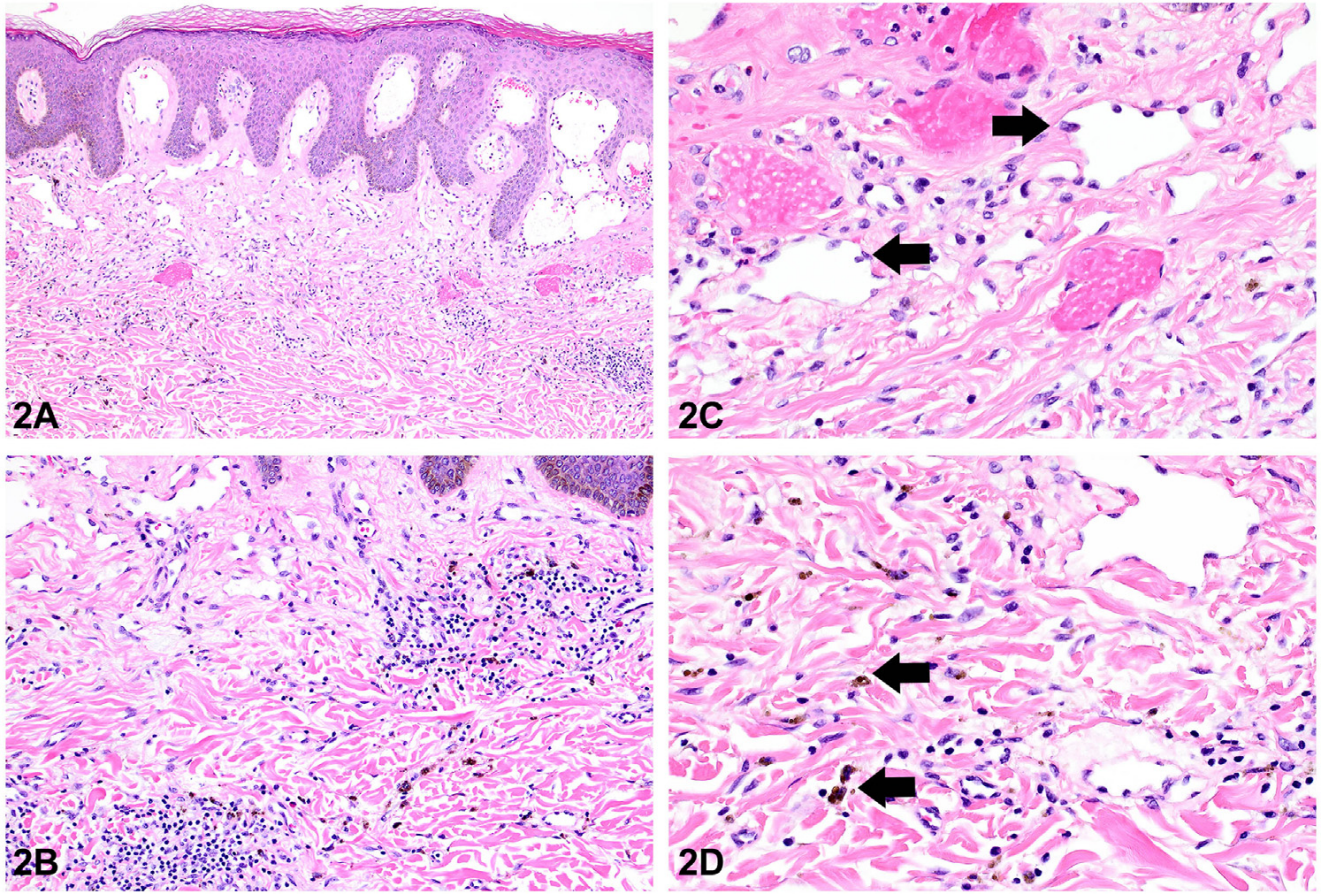

A 34-year-old female with a history of metastatic breast cancer in remission after treatment presented with an asymptomatic, hyperpigmented papule on her central abdomen. The patient reported that the lesion had been present for a few months, suddenly darkened on a single day during this period, and then remained stable. Review of systems was negative for any systemic findings. Physical exam showed an approximately 1 cm purplish-blue papulonodule with surrounding hyperpigmentation (Fig 1). The patient reported minimal tenderness in the surrounding area. The papule was excised, and histological findings are shown (Fig 2).

**Question 1: What is the most likely diagnosis?**

A. Sister Mary Joseph nodule
B. Angiosarcoma
C. Targetoid hemosiderotic hemangioma (THH)
D. Cutaneous sarcoidosis
E. Kaposi sarcoma (KS)

**Answers:**

A. Sister Mary Joseph nodule – Incorrect. Although a new cutaneous papule in a patient with known prior metastatic breast cancer should raise concern for a cutaneous metastasis such as a Sister Mary Joseph nodule, the targetoid appearance and the pathology are most consistent with THH.^1^
B. Angiosarcoma – Incorrect. Angiosarcomas are typically purplish-red, have irregular borders, exhibit surface bleeding, and would be expected to show ongoing progression. These findings are not present in this patient.
C. Targetoid hemosiderotic hemangioma (THH) – Correct. THH has a classic, clinical presentation of a purplish papule surrounded by an ecchymotic/brown ring that expands or fades while leaving the central papule intact.^2^ The papule may darken over time as the patient reported.
D. Cutaneous sarcoidosis – Incorrect. While cutaneous sarcoidosis can present as a hyperpigmented papule, the targetoid appearance would not be expected, and granulomas would be expected to be seen on histopathology.
E. Kaposi sarcoma (KS) – Incorrect. Although KS can appear dark brown, KS typically presents as a multifocal vascular neoplasm with a distinct histopathology.

**Question 2: Which histological finding is most characteristic of this lesion?**

A. Hobnail endothelial cells
B. Irregular, anastomosing vascular channels
C. Infiltrative spindle cells
D. Epidermal necrosis
E. Atypical melanocytes

**Answers:**

A. Hobnail endothelial cells – Correct. THH is a wedge-shaped vascular proliferation (Fig 2, *A*) characterized by vessels lined by bland endothelial cells (Fig 2, *B*) with plump nuclei projecting into the lumina, conferring a hobnail appearance (Fig 2, *C*; arrows).^3^ Accordingly, THH is also referred to as a hobnail hemangioma. Hemorrhage and hemosiderin deposition are usually prominent features (Fig 2, *D*; arrows), whereas inflammation is generally absent.
B. Irregular, anastomosing vascular channels – Incorrect. Angiosarcoma is characterized by an infiltrative, poorly circumscribed tumor composed of irregular, anastomosing vascular channels with atypical endothelial cells showing multilayering, nuclear atypia, and often conspicuous mitotic activity.
C. Infiltrative spindle cells – Incorrect. KS is a vascular proliferation caused by human herpesvirus 8 (HHV-8), which shows irregular slit-like vascular channels and infiltrative spindle cells with associated hemorrhage and often scattered plasma cells.
D. Epidermal necrosis – Incorrect. Epidermal necrosis would be expected in the setting of tissue ischemia or a severe cutaneous adverse drug reaction.
E. Atypical melanocytes – Incorrect. Atypical melanocytes would be expected in the context of pigmented neoplasms such as atypical nevi or melanoma.

**Question 3: This lesion is most likely to stain positively for which of the following immunohistochemical stains?**

A. HHV8
B. SOX10
C. Cytokeratin 7
D. CD31
E. p63

**Answers:**

A. HHV8 – Incorrect. Nuclear HHV8 latency-associated nuclear antigen-1 is both sensitive and specific for KS. The tumor cells in KS are also positive for endothelial markers, such as CD31, CD34, and ETS-related gene (ERG).
B. SOX10 – Incorrect. SOX10 is expressed by melanocytes and would be expected to be positive in nevi and most melanomas.
C. Cytokeratin 7 – Incorrect. CK7 is a cytoplasmic marker with expression in many normal epithelia and epithelial tumors, including primary extramammary Paget disease and Paget disease of the breast.^4^ Histopathology would show an intraepithelial proliferation of large pale epithelioid cells.
D. CD31 – Correct. CD31 is an abundant membrane glycoprotein found on vascular endothelium and is a highly sensitive and specific endothelial immunohistochemical marker, confirming the vascular origin of THH when combined with the clinical presentation.^5^
E. p63 – Incorrect. p63 is a transcription factor associated with epidermal keratinocyte proliferation and epidermal growth. In the skin, p63 expression is typically associated with cutaneous squamous cell carcinoma, basal cell carcinoma, and primary cutaneous adnexal neoplasms.^4^ Squamous cell carcinoma often presents as a pink-red hyperkeratotic papule, unlike our patient’s pigmented and smooth papule.

## Conflicts of interest

None disclosed.

## References

[1] Cohen P.R. (2021). Pleomorphic appearance of breast cancer cutaneous metastases. Cureus.

[2] Yoon S.Y., Kwon H.H., Jeon H.C., Lee J.H., Cho S. (2011). Congenital and multiple hobnail hemangiomas. Ann Dermatol.

[3] Mentzel T., Partanen T.A., Kutzner H. (1999). Hobnail hemangioma (“targetoid hemosiderotic hemangioma”): clinicopathologic and immunohistochemical analysis of 62 cases. J Cutan Pathol.

[4] Wieland R., Adhikari P., North J. (2023). The utility of p63, CK7, and CAM5.2 staining in differentiating pagetoid intraepidermal carcinomas. J Cutan Pathol.

[5] DeYoung B.R., Swanson P.E., Argenyi Z.B. (1995). CD31 immunoreactivity in mesenchymal neoplasms of the skin and subcutis: report of 145 cases and review of putative immunohistologic markers of endothelial differentiation. J Cutan Pathol.

